# Supplementary material for: Impacts of the quinone-functionalized biochar on anaerobic digestion: Beyond the redox property of biochar
Source: PLoS One. 2025 Apr 22;20(4):e0322275. doi: 10.1371/journal.pone.0322275 (PMC12013935; doi:10.1371/journal.pone.0322275)
Supplement: S1 File — (DOCX) [file pone.0322275.s001.docx]

**Supporting Information for**

***Impacts of the quinone-functionalized biochar on anaerobic digestion: Beyond the redox property of biochar***

Qian Jiang^1,2*^, Wentao Zhou^1^, Yue Chen^1^, Zhenglong Peng^1^, Chengcheng Li^1^

^1^ School of Biological and Materials Engineering, Suqian University, Suqian, China

^2^ Jiangsu Engineering Research Center of Novel Functional Film and Technology, Biological and Materials Engineering, Suqian University, Suqian, China

^*^ Corresponding author

E-mail: jiangqian@squ.edu.cn (QJ)

# Materials and methods

## Characterization of biochar

For pH measurement, algal biochar (ABC) was added into ultrapure water at a ratio of 1:100 (w/w). Then biochar suspensions were mixed at 130 rpm for 1h in an incubator shaker and measured using a pH meter (Mettler Toledo, Switzerland). The specific surface area (SSA) of ABC was measured by N_2_ adsorption isotherms at 77 K with the Brunauer-Emmett-Teller (BET) method (Belsorp-Max, Japan). The iodine value and phenol adsorption value of ABC were measured according to the national standard methods of China (GB/T12496.8-2015 and GB/T7702.8-2008, respectively). The particle size distribution of ABC was determined with a laser particle size analyzer (BT-2003, China).

## Measurement of the redox activity of biochar materials

Biochar powders were immobilized on carbon paper to test their redox properties by cyclic voltammetry (CV) method. Specifically, 10 mg biochar powder was dispersed in 1 mL 5% Nafion solution with a vortex. Next, 20 μL of the resulting dispersion was dropped onto the center of carbon paper (1×1 cm), and then all the biochar-immobilized carbon papers were dried carefully at 60 ^o^C in an oven for 8 h. CV measurement was performed in a designed three-electrode electrochemical cell (working volume 10 mL) using an electrochemical workstation (CHI660E, Chenhua, China). The biochar-immobilized carbon paper, a glassy carbon electrode, and a saturated KCl silver/silver chloride were used as working, counter, and reference electrodes, respectively. A buffer solution (0.1 mol/L KCl and 0.1 mol/L phosphates, pH=7) was used as the supporting electrolyte. Before measurements, N_2_ was used to purge oxygen from the solution and maintain the anaerobic conditions of the measurement processes. The scan direction for the CV was changed from reduction to oxidation and the scan rate was 20 mV/s.

## Quantitation of the electron exchange capacity of biochar

A designed three-electrode cell (working volume 10 mL) with a graphite rod electrode, a glassy carbon electrode, and a saturated KCl silver/silver chloride were used as working, counter, and reference electrodes, respectively. Measurements of EEC was performed in a buffer solution (0.1 mol/L KCl and 0.1 mol/L phosphates, pH 7) with an electrochemical workstation (CHI660E, Chenhua Co., Shanghai, China). The applied potential for the EAC and EDC quantitation was +0.61 V and -0.49 V (vs SHE), respectively. The electron transfer shuttle of the EAC and EDC measurements was 2,2’-azonio-bis (3-ethylbenzothiazoline-6-sulfonic acid) diammonium salt (ABTS, Sigma) and diquat dibromide monohydrate (DQ, Macklin), respectively. N_2_ flow was employed to maintain anaerobic conditions before the measurement. After certain volumes of 10 g/L biochar suspensions were added, an I-t model was performed, and then EAC and EDC could be calculated according to the integration results of current peaks in the I-t curve. The I-t curves were fitted and calculated using Origin software (Origin Pro 9.5, USA).

## Model fitting analysis for methane production

The methane production process was fitted and analyzed by the modified Gompertz model:

$P_{(t)}=P_{m}\cdot exp\left\{ -exp\left[ \frac{R_{m}\cdot e}{P_{m}}\left( \lambda-t \right)+1 \right] \right\}$ (1)

where P_(t)_ is the cumulative methane production at time t, mL/g COD; P_m_ is the maximum methane production potential, mL/g COD; R_m_ is the maximum methane production rate (MPR), mL/g COD d; λ is the lag phase time, d; t is the duration time, d; e is constant (2.71). The methane production curve and the relevant parameters were simulated using Origin Pro 9.5 (Origin Lab Corporation, MA, US).

**Table S1.** The main characteristics of different biochar materials used in this study

|  | **pH** | **Particle size**  **(μm)** | **SSA**  **(m^2^/g)** | **Iodine value (mg/g)** | **Phenol adsorption value (mg/g)** |
| --- | --- | --- | --- | --- | --- |
| ABC | 7.3 ± 0.1 | 218.8 ± 56 | 145.6 ± 39 | 289.8 ± 87 | 101.2 ± 26 |
| ABC-AQS | 6.1 ± 0.3 | 165.3 ± 38 | ND^a^ | ND^a^ | ND^a^ |

^a^ ND: not detected in this study
