# Supplementary material for: Impacts of the quinone-functionalized biochar on anaerobic digestion: Beyond the redox property of biochar
Source: PLoS One. 2025 Apr 22;20(4):e0322275. doi: 10.1371/journal.pone.0322275 (PMC12013935; doi:10.1371/journal.pone.0322275)
Supplement: Attachment — Submitted filename: Comments.pdf [file pone.0322275.s003.pdf]

Manuscript Number: PONE-D-25-01514

Title: Impacts of the quinone-functionalized biochar on anaerobic digestion: Beyond the redox property of biochar

In this manuscript, anthraquinone-2-sulfonate (AQS) was immobilized on algal biochar (ABC) by surface doping method, and the impacts of the quinone-functionalization process on algal biochar for regulating methane production were investigated. Furthermore, the authors have explored the mechanisms associated with the methanogenic performances with the addition of quinone-functionalized biochar (ABC-AQS). Interestingly, the study's findings will help to clarify the purpose and importance of the quinone-functionalization process on algal biochar in controlling methane generation, which will be helpful in the development of designed biochar for real-world uses. Overall, this is a meticulous and informative work, which is a topic of interest to the researchers in the related areas. Although the work is well designed and experiments are well organized, however, some major concerns have arisen on reading the manuscript which should be addressed.

1. The abstract is not well-organized. Regarding the abstract, it should be noted that a good abstract should contain the following points:
    - The state of the field and/or the gap your research is filling
    - Describing what you did to develop your argument
    - The summary of major findings
    - The larger implications of your findings

    In the abstract of the current manuscript, the authors have mainly described what they have done and the summary of their major findings. However, the other three points are missing in the current abstract. In this aspect, the abstract is not compelling. The authors are advised to organize the abstract by discussing the above-mentioned points one by one and highlight the significance/novelty of the work. Additionally, for further improvement of the Abstract reach, I suggest that you incorporate a note in your end statement, commenting on the novelty of the work.
2. In the introduction part, please include a schematic diagram in order to get an in-depth knowledge on the purpose of the current study. It will further strengthen the novelty of the current study.
3. Statistical analysis is missing in Figure 2(b) and (c). The authors are strongly recommended to include error bars in the mentioned Figures.
4. Materials and Methods: Some experimental details are missing in the current manuscript. Various techniques described as cyclic voltammetry (CV) method and X-ray diffraction measurements should be described in detail with different parameters used for the analysis. What is the concentration used for those analysis? What is the solvent used for these studies? Please mention all these things in the methods.
5. For completeness purposes, please include a comparison of the results reported in this work and others.

6. The authors are advised to briefly address the future perspectives, limitations, and drawbacks of the current study in the conclusion part. It is not necessary to go into great depth when describing the scope of future study; a rough sketch of the areas needing additional research and/or optimization should do.
7. Please provide a descriptive supporting information section in the manuscript with at least 2-3 sentences.
