# Supplementary material for: Impacts of the quinone-functionalized biochar on anaerobic digestion: Beyond the redox property of biochar
Source: PLoS One. 2025 Apr 22;20(4):e0322275. doi: 10.1371/journal.pone.0322275 (PMC12013935; doi:10.1371/journal.pone.0322275)
Supplement: Attachment — Submitted filename: PONE-D-25-01514_reviewer.docx [file pone.0322275.s002.docx]

PONE-D-25-01514_reviewer

Impacts of the quinone-functionalized biochar on anaerobic digestion: Beyond the

redox property of biochar

The manuscript demonstrates a highly relevant issue related to attempts of intensification of biogas production using engineered biochar. It was hypothesized that quinone functional groups could intensify methane production due to increase of redox potential of biochar. Results showed opposite influence on the overall methane production meaning its inhibition, but acidogenesis rate was improved. Previous results were presented in the Introduction section including the description of knowledge gaps. Detailed protocols and methods for chemical analysis and measurements are presented as supporting information online. Details of the methodology are sufficient to allow the experiments to be reproduced. Units of measurement were clearly defined in all tables and figures. P-values were reported for parameters of electron capacity of different types of biochar (p < 0.01). Displaying data in plots is sufficient.

At the same time, I came up with some major and minor concerns.

1. Section Introduction is well-designed for reader without specific knowledge in this area, but statement of hypothesis is lack.
2. Authors claimed accepting of PLOS’s Data Availability policy, but I could find dataset of experiment’s results in SI, and original data are not deposited in appropriate repositories.
3. Section Materials and Methods was very well described, but a paragraph about Statistical analysis should be developed. It is not clear from the text which measures of variance (standard deviation, standard error of the mean, confidence intervals) and central tendency (mean, median) were being presented.
4. Presented results related to biochar properties and methane production are relevant and important, but no regression analyses or relationships between those parameters were found.
5. There is a lack of argumentation and discussion about the choice of substrate for biochar production and its ecological safety due to waste origin and probability of containing some pollutants (line76).
6. The number of samples for analysis was not found in the manuscript.
7. It was stated that glucose served as the sole carbon source for the anaerobic digestion test to avoid possible interference from substrates (line 110), but it’s not clear if the same results of methane production will be obtained in the case of real-stated biogas reactor with common substrate.
8. In the Model fitting analysis paragraph reference to SI was stated as well as series of other reports. Which reports have been meant?
9. Sections Results and Discussion are combined, but it will be easier to read manuscript if they are separated.
10. Obtained results are explained and discussed, but practical implications are lacking as well as further research due to the rejecting of initial assumptions about the influence of modified biochar on methane production.
11. The manuscript is well organized but written not enough clearly to be accessible to non-specialists.

However, the study itself shows sufficient potential that the authors are encouraged to resubmit a revised version according to mentioned comments.
