# Supplementary material for: Impacts of the quinone-functionalized biochar on anaerobic digestion: Beyond the redox property of biochar
Source: PLoS One. 2025 Apr 22;20(4):e0322275. doi: 10.1371/journal.pone.0322275 (PMC12013935; doi:10.1371/journal.pone.0322275)
Supplement: Attachment — Submitted filename: Response to Reviewers.docx [file pone.0322275.s004.docx]

**Response to Reviewers**

**Manuscript Number:** PONE-D-25-01514
**Manuscript Title:** Impacts of the quinone-functionalized biochar on anaerobic digestion: Beyond the redox property of biochar
**Article Type:** Research Article

**Author(s):** Qian Jiang, Wentao Zhou, Yue Chen, Zhenglong Peng, Chengcheng Li

----------------------------------------------------------------------------------------------------------

**Journal Requirements:**

1. *Please ensure that your manuscript meets PLOS ONE's style requirements, including those for file naming. The PLOS ONE style templates can be found at*

*https://journals.plos.org/plosone/s/file?id=wjVg/PLOSOne_formatting_sample_main_body.pdf and*

*https://journals.plos.org/plosone/s/file?id=ba62/PLOSOne_formatting_sample_title_authors_affiliations.pdf*

**Response:** Thanks for the comments. The manuscript has been carefully revised according to the PLOS ONE style templates:

The tittle, author, affiliations formatting of the manuscript have been revised, please see Page 1, Lines 1-11; the same revisions have also been made in the “Supporting Information” file.

The manuscript body formatting has been revised, please see Page 3, Lines 35-36, 39, 42-45; Page 4, Line 59, 68; Page 5, Line 88; Page 6, Lines 106-108; Page 11, Lines 202-203; Page 12, Line 238; Page 13, Lines 240-241, 253-255, 259; Page 14, Line 275; Page 15, Lines 288-289; Page 16, Line 307; the file naming has been revised according to the guidelines.

2. *In your Methods section, please provide additional information regarding the permits you obtained for the work. Please ensure you have included the full name of the authority that approved the field site access and, if no permits were required, a brief statement explaining why.*

**Response:** Thanks for the comments. No permits were required in this study and explanations were listed as follows: the blue algae biomass was collected by the filtration method, which has been performed by the authors of this study; the preparation of biochar was performed in a lab-scale tube furnace (Shengli Instruments, China), the modification of biochar, the batch anaerobic digestion tests, and the chemical analysis were all performed in the laboratory. Accordingly, the additional information about the “Materials and methods” was revised, please see Page 5, Lines 82-87.

3. *Thank you for stating the following financial disclosure:*

*“Suqian Sci&Tech Program (Grant NO. K202333), Scientific Research Foundation of Suqian University (NO.106-CK00042/060)”*

*Please state what role the funders took in the study. If the funders had no role, please state: "The funders had no role in study design, data collection and analysis, decision to publish, or preparation of the manuscript."*

*If this statement is not correct you must amend it as needed.*

*Please include this amended Role of Funder statement in your cover letter; we will change the online submission form on your behalf.*

**Response:** Thanks for the comments. The financial disclosure was stated in the cover letter: “The funders had no role in study design, data collection and analysis, decision to publish, or preparation of the manuscript.” Please see the cover letter.

4. *Thank you for stating the following in the Acknowledgments Section of your manuscript:*

*“The preparation of algal biochar and anaerobic digestion test were performed at Jiangsu key laboratory of anaerobic biotechnology, Jiangnan University. This study was financially supported by Suqian Sci&Tech Program (Grant NO. K202333), Scientific Research Foundation of Suqian University (NO.106-CK00042/060). “*

*We note that you have provided funding information that is currently declared in your Funding Statement. However, funding information should not appear in the Acknowledgments section or other areas of your manuscript. We will only publish funding information present in the Funding Statement section of the online submission form.*

*Please remove any funding-related text from the manuscript and let us know how you would like to update your Funding Statement. Currently, your Funding Statement reads as follows:*

*“Suqian Sci&Tech Program (Grant NO. K202333), Scientific Research Foundation of Suqian University (NO.106-CK00042/060)”*

*Please include your amended statements within your cover letter; we will change the online submission form on your behalf.*

**Response:** Thanks for the comments. The funding-related text has been removed from the Acknowledgments section of manuscript. The Acknowledgments section has been revised to “The preparation of algal biochar and anaerobic digestion test were performed at Jiangsu key laboratory of anaerobic biotechnology, Jiangnan University. Thanks for the assistance of He Liu and Qihao Cao.” Please see Page 17, lines 337-338.

Funding Statement has been revised to “Funding: This research is financially supported by the Suqian Sci&Tech Program (Grant NO. K202333) and the Scientific Research Foundation of Suqian University (NO.106-CK00042/060). The funders had no role in study design, data collection and analysis, decision to publish, or preparation of the manuscript.” Please see the cover letter.

5. *We note that Figure 1 in your submission contain copyrighted images. All PLOS content is published under the Creative Commons Attribution License (CC BY 4.0), which means that the manuscript, images, and Supporting Information files will be freely available online, and any third party is permitted to access, download, copy, distribute, and use these materials in any way, even commercially, with proper attribution. For more information, see our copyright guidelines: http://journals.plos.org/plosone/s/licenses-and-copyright.*

*We require you to either (1) present written permission from the copyright holder to publish these figures specifically under the CC BY 4.0 license, or (2) remove the figures from your submission:*

*a. You may seek permission from the original copyright holder of Figure 1 to publish the content specifically under the CC BY 4.0 license.*

*We recommend that you contact the original copyright holder with the Content Permission Form (http://journals.plos.org/plosone/s/file?id=7c09/content-permission-form.pdf) and the following text:*

*“I request permission for the open-access journal PLOS ONE to publish XXX under the Creative Commons Attribution License (CCAL) CC BY 4.0 (http://creativecommons.org/licenses/by/4.0/). Please be aware that this license allows unrestricted use and distribution, even commercially, by third parties. Please reply and provide explicit written permission to publish XXX under a CC BY license and complete the attached form.”*

*Please upload the completed Content Permission Form or other proof of granted permissions as an "Other" file with your submission.*

*In the figure caption of the copyrighted figure, please include the following text: “Reprinted from [ref] under a CC BY license, with permission from [name of publisher], original copyright [original copyright year].”*

*b. If you are unable to obtain permission from the original copyright holder to publish these figures under the CC BY 4.0 license or if the copyright holder’s requirements are incompatible with the CC BY 4.0 license, please either i) remove the figure or ii) supply a replacement figure that complies with the CC BY 4.0 license. Please check copyright information on all replacement figures and update the figure caption with source information. If applicable, please specify in the figure caption text when a figure is similar but not identical to the original image and is therefore for illustrative purposes only.*

**Response:** Thanks for the comments. Figure 1 has been removed from the manuscript, and the detailed information for the preparation of algal biochar in the removed figure has been added and revised in the manuscript, please see Page 5, Lines 82-90.

For the rest of figures, captions and citations were all revised accordingly. Please see Page 5, line 91; Page 6, lines 98, 106-108; Page 9, line 175; Page 10, Lines 177, 179, 185, 192-194; Page 11, lines 200-203, 207, 210, 213; Page 12, lines 220, 223-225, 233, 235; Page 13, lines 240-241, 244, 246, 247, 253-255; Page 14, line 277; Page 15, lines 288-289, 292, 294, 314.

**Reviewer’s comments:**

**Reviewer #1:**

**1. General comments**

*Reviewer #1: The manuscript demonstrates a highly relevant issue related to attempts of intensification of biogas production using engineered biochar. It was hypothesized that quinone functional groups could intensify methane production due to increase of redox potential of biochar. Results showed opposite influence on the overall methane production meaning its inhibition, but acidogenesis rate was improved. Previous results were presented in the Introduction section including the description of knowledge gaps. Detailed protocols and methods for chemical analysis and measurements are presented as supporting information online. Details of the methodology are sufficient to allow the experiments to be reproduced. Units of measurement were clearly defined in all tables and figures. P-values were reported for parameters of electron capacity of different types of biochar (p < 0.01). Displaying data in plots is sufficient.*

*At the same time, I came up with some major and minor concerns.*

**Response:** Thanks for the comments. The manuscript has been carefully revised according to the comments. The details of the revisions have been listed below following each specific comment, point-by-point. The response to each comment is in blue in this file; all of the errors as we know have been corrected and marked with the red color in the revised manuscript.

**2. Special comments**

*1. Section Introduction is well-designed for reader without specific knowledge in this area, but statement of hypothesis is lack.*

**Response:** Thanks for the comment. The section Introduction of this manuscript has been revised, and the statement of hypothesis has been added. Please see Page 4, Lines 63-70.

*2. Authors claimed accepting of PLOS’s Data Availability policy, but I could find dataset of experiment’s results in SI, and original data are not deposited in appropriate repositories.*

**Response:** Thanks for the comment. The authors confirm that all data underlying the findings are within the paper and its Supporting Information files. In the data statement we have described where the data may be found in full sentences: All relevant data are within the manuscript and its Supporting Information files. Thanks again for your suggestion.

*3. Section Materials and Methods was very well described, but a paragraph about Statistical analysis should be developed. It is not clear from the text which measures of variance (standard deviation, standard error of the mean, confidence intervals) and central tendency (mean, median) were being presented.*

**Response:** Thanks for the comment. The description of the statistical analysis of data in this study has been revised, please see in the section Materials and Methods, Page 9, Lines 166-171.

*4. Presented results related to biochar properties and methane production are relevant and important, but no regression analyses or relationships between those parameters were found.*

**Response:** Thanks for the comment. Yes, we all agree that biochar properties and methane production are relevant and important. However, results in this study have showed opposite influence on the overall methane production by the modified biochar, therefore the regression analyses or relationships between biochar properties and methanogenic improvement were not performed. Instead the main purpose of this study was to clarify the role and significance of the quinone-functionalization process on algal biochar in controlling methane generation, the study's findings will help to the development of designed biochar for real-world uses. To make it clear, the implications of this work has been added and revised, pleased see Page 17, Lines 332-334.

*5. There is a lack of argumentation and discussion about the choice of substrate for biochar production and its ecological safety due to waste origin and probability of containing some pollutants (line76).*

**Response:** Thanks for the comment. **Firstly,** Taihu blue algae, a kind of abundant and harmful biomass waste in the water treatment of Wuxi, China. Till 2018, the yearly refloated Taihu blue algae reached about 1.87 million tons, while the refloated Taihu blue algae of Wuxi city since 2007 totaled about 13.43 million tons, consisting 90% of the algal amounts refloated in Jiangsu Province, China. It is of great important significance to develop an approach for the treatment and disposal of the Taihu blue algae biomass;

**Secondly,** the preparation of biochar from the Taihu blue algae has been consistently studied by our team, a series of works has been published in the nearly 5 years, eg., 10.1016/j.biortech.2021.125493; 10.1016/j.enconman.2022.115417; 10.1016/j.jece.2023.109850; 10.1016/j.jwpe.2024.106123. Moreover, algal biomass originated biochar were found to possess significantly higher N-content than that from the lignocellulosic biomass, which would in turn promote the electrochemical traits for the electron transfer during the biochemical process. Therefore, the Taihu blue algae was continued to serve as the precursor for biochar production in this study, hoping to further expand the treatment and disposal of this solid waste;

**Lastly,** according to our previous study, as well as other study (Shi et al., 2015, Environmental science & Technology), the dominated community of the Taihu blue algal biomass obtained was *Microcystis*; moreover, algal biomass was collected from the algal blooming sites alongside Taihu Lake of Wuxi city, filtration and cold drying method were employed to avoid chemicals addition during the biomass harvest; the obtained algae powder were stored at 4 ^o^C before use. Therefore, we confirm that the probability of some pollutants being present in algal biomass should be low in this study.

Based on the above consideration, the description the choice of substrate for biochar production in this manuscript has been added and revised, please see Page 5, Lines 82-90.

*6. The number of samples for analysis was not found in the manuscript.*

**Response:** Thanks for the comment. The description of samples for analysis in this study could be found in the section Materials and methods and the section Results and discussion:

(1) The anaerobic digestion tests were conducted in eight customized glass reactors (1.2 L) with working volume of 600 mL, both the gas and liquid samples were analyzed during the batch experiments. After first batch of anaerobic digestion, the second batch of anaerobic digestion was repeated under the same condition as the first batch. The resulted data obtained from the above two batch experiments were analyzed and plotted in Figure 2 of this study. Please see Page 8, Lines 145-148;

(2) For the analysis of biochar samples, inclduing pH, CV, and electron exchange capacity (EEC), the number of samples were set to a minimum of 3 and the resultant data was showed in the Figures and the S1_Table. For example, in Figure 4, the number of samples for quantitative EEC analysis was 6, the raw data point and the statistical analysis results were displayed directly in the image.

*7. It was stated that glucose served as the sole carbon source for the anaerobic digestion test to avoid possible interference from substrates (line 110), but it’s not clear if the same results of methane production will be obtained in the case of real-stated biogas reactor with common substrate.*

**Response:** Thanks for the comment. Yes, we all agree that glucose served as the sole carbon source for the anaerobic digestion test to avoid possible interference from substrates. This is because the main purpose of this study was to explore the impact of modified biochar, for which the effect of other factors in the anaerobic reactors needed to be minimized as much as possible. This strategy has also been adopted by other researchers, eg., http:// doi. 10.1021/acs.est.0c00112.

Moreover, we are also very interested in the impacts of quinone-functionalized biochar on the real-stated biogas reactor with common substrate. The mechanisms and feasibility of the addition of functionalized biochar to improve the anaerobic digestion performance are still worthy of further investigation, and our future work will consistently pay attention to this area. Thank you again for your insightful comments and suggestions.

*8. In the Model fitting analysis paragraph reference to SI was stated as well as series of other reports. Which reports have been meant?*

**Response:** Thanks for the comment. Done. The reference for the Model fitting analysis in this study has been added, and the subsequent reference numbers have been revised accordingly. Please see Page 8, Line 156; Page 21-23, Lines 428-454.

*9. Sections Results and Discussion are combined, but it will be easier to read manuscript if they are separated.*

**Response:** Thanks for the comment. We believe that the combination of the sections Results and Discussion will help readers gain a quick and in-depth understanding of the results and its implications of this study. Besides, this approach has also been applied in the other published articles of PLOS One, e.g., 10.1371/journal.pone.0113888; 10.1371/journal.pone.0137467; 10.1371/journal.pone.0129448. The combination of the sections Results and Discussion should be also acceptable.

*10. Obtained results are explained and discussed, but practical implications are lacking as well as further research due to the rejecting of initial assumptions about the influence of modified biochar on methane production.*

**Response:** Thanks for the comment. In this study we hypothesized that the redox activity of biochar could be enhanced by immobilizing quinoid functional groups on its surface, and result showed that the redox activity of biochar was significantly after modification, especially the electron donating capacity of biochar was increased by 113.5% compared to the raw biochar.

However, variation in biochar properties was found to have an interesting effect on the subsequent anaerobic digestion process: **On one hand,** the acidogenesis rate during anaerobic digestion test was improved by 26.3% with the addition of the functionalized biochar, indicating that the enhanced redox activity of biochar was favorable for the microbial metabolism during anaerobic digestion; **On the other hand**, the methanogenesis stage of anaerobic digestion was found to be inhibited by the modified biochar. Combining the experimental phenomena in this study and previous works, we believed that the related mechanisms originated from the strong acid treatment stage involved in the biochar modification process. Compared to the improvement of redox activity after biochar modification, the microbial inhibition caused by side reactions during chemical modification may have a stronger impact on anaerobic digestion process.

Given the fact that most studies in the field of biochar modification have focused on the removal of organic and inorganic pollutants from water, soil, and other systems, the knowledge of engineered biochar on anaerobic digestion process is worthy of further exploration. The findings of this study give us an important clue that when designing a biochar modification procedure for a given biotechnological process, the possible influences of chemical side reactions during biochar modification process on subsequent microbial metabolism should be emphasized.

Based on the above consideration, the implications of this work has been added and revised, pleased see Page 14, Lines 278-282; Page 16, Lines 320-322; Page 17, Lines 332-334.

*11. The manuscript is well organized but written not enough clearly to be accessible to non-specialists. However, the study itself shows sufficient potential that the authors are encouraged to resubmit a revised version according to mentioned comments.*

**Response:** Thanks for the comment. The manuscript has been carefully revised according to the comments. The details of the revisions have been listed following each specific comment, point-by-point. All of the errors as we know have been corrected and marked with the red color in the revised manuscript. Thank you again for your comments and suggestions.

**Reviewer #2:**

**1. General comments**

*In this manuscript, anthraquinone-2-sulfonate (AQS) was immobilized on algal biochar (ABC) by surface doping method, and the impacts of the quinone-functionalization process on algal biochar for regulating methane production were investigated. Furthermore, the authors have explored the mechanisms associated with the methanogenic performances with the addition of quinone-functionalized biochar (ABC-AQS). Interestingly, the study's findings will help to clarify the purpose and importance of the quinone-functionalization process on algal biochar in controlling methane generation, which will be helpful in the development of designed biochar for real-world uses. Overall, this is a meticulous and informative work, which is a topic of interest to the researchers in the related areas. Although the work is well designed and experiments are well organized, however, some major concerns have arisen on reading the manuscript which should be addressed.*

**Response:** Thanks for the comments. The manuscript has been carefully revised according to the comments. The details of the revisions have been listed below following each specific comment, point-by-point. The response to each comment is in blue in this file; all of the errors as we know have been corrected and marked with the red color in the revised manuscript.

**2. Special comments**

*1. The abstract is not well-organized. Regarding the abstract, it should be noted that a good abstract should contain the following points:*

*• The state of the field and/or the gap your research is filling*

*• Describing what you did to develop your argument*

*• The summary of major findings*

*• The larger implications of your findings*

*In the abstract of the current manuscript, the authors have mainly described what they have done and the summary of their major findings. However, the other three points are missing in the current abstract. In this aspect, the abstract is not compelling. The authors are advised to organize the abstract by discussing the above-mentioned points one by one and highlight the significance/novelty of the work. Additionally, for further improvement of the Abstract reach, I suggest that you incorporate a note in your end statement, commenting on the novelty of the work.*

**Response:** Thanks for the comment. The abstract of this manuscript has been revised according to the suggestion, details could be listed as follows:

For the state of the field and/or the gap your research is filling: biochar was modified to improve its performance during environmental applications, however, the impacts of engineered biochar on a given biotechnological process (e.g., anaerobic digestion) is still limited. Therefore, this knowledge gap was highlighted at the beginning of the Abstract, and the related description in this manuscript has been revised to “Recent developments in biochar materials have led to a renewed interest in biochar modification for environmental applications, however, much uncertainty still exists about the impact of engineered biochar on a given biotechnological process”, please see Page 2, lines 16-18;

For the statement of novelty and significance of this study, the related contents in the end of abstract has been revised to “The findings of this study give us an important clue that when designing a biochar modification procedure for anaerobic digestion, attentions should be paid to the possible influences of chemical side reactions during biochar modification process on the subsequent microbial metabolism, which would be valuable in designing engineered biochar for practical applications”, please see Page 2, lines 28-31. Thanks again for your suggestion.

*2.* *In the introduction part, please include a schematic diagram in order to get an in-depth knowledge on the purpose of the current study. It will further strengthen the novelty of the current study.*

**Response:** Thanks for the comment. The introduction part of this work has been revised and divided into five paragraphs:

Firstly, the role and significance of anaerobic digestion technology were summarized; In the second paragraph, the improvement of anaerobic digestion based on the addition of biochar materials was introduced and key material properties related to the enhancement of anaerobic digestion was highlighted; Then in the third paragraph, previous reports using biochar modification to improve the redox activity of biochar were summarized, focusing on the previous studies of using quinone-based modification to improve the redox activity of biochar;

Based on the above consideration, the statement of hypothesis in the section Introduction of this manuscript has been revised: the redox capacity of biochar could be enhanced by immobilizing quinone groups on its surface, and the efficiency of anaerobic digestion should be improved by the quinone-functionalized biochar; and the aim and significance of this work have been summarized in this last of the section Introduction.

We believe that the readability of the manuscript could be enhanced by the revision of introduction section, especially the revision of the research hypothesis could help readers to get an in-depth knowledge on the purpose of this study. Thanks again for your suggestion. Revisions in the section Introduction of this manuscript could be found in Page 4, Lines 63-70.

*3.* *Statistical analysis is missing in Figure 2(b) and (c). The authors are strongly recommended to include error bars in the mentioned Figures.*

**Response:** Thanks for the comment. Done, statistical analysis has been performed and error bars in Figure 2(b) and (c) was easily overlooked due to the thickness of the lines and the size of the plot points in the mentioned figures. Figure 2 has been revised, please see the uploaded figure file (Fig 1).

*4.* *Materials and Methods: Some experimental details are missing in the current manuscript. Various techniques described as cyclic voltammetry (CV) method and X-ray diffraction measurements should be described in detail with different parameters used for the analysis. What is the concentration used for those analysis? What is the solvent used for these studies? Please mention all these things in the methods.*

**Response:** Thanks for the comment. The experimental details about X-ray diffraction measurements has been revised from “The crystalline structure of biochar was determined by X-ray diffraction (XRD, Bruker, Germany)” to “The crystalline structure of biochar sample was examined by X-ray diffraction (XRD, D2 Phaser, Bruker, Germany) over a 2θ collection range of 10^o^ - 80^o^”, please see Page 6, Lines 115-116.

The experimental details about cyclic voltammetry (CV) method (including solvent and concentration for analysis, electrodes, scan direction, and scan rate) were all described in the section “Materials and methods” of Supporting Information, titled “Measurement of the redox activity of biochar materials”, Please see the Supporting Information file. Moreover, the related description in manuscript has been revised, please see Page 7, Lines 117-119.

The experimental details for quantitation of electron exchange capacity of biochar sample (including solvent and concentration for analysis, electrodes, electrochemical workstation, and EEC calculation) were all described in the section “Materials and methods” of Supporting Information, titled “Quantitation of the electron exchange capacity of biochar”, Please see the Supporting Information file. Moreover, the related description in manuscript has been revised, please see Page 7, Lines 117-119.

In addition, the description of measurement of raw biochar samples has been revised to “More experimental details about the measurement of raw biochar samples, including pH, particle size, and specific surface area, were summarized in the section “Materials and methods” of Supplementary Information, please see page 5, lines 88-90; the description of model fitting analysis in this study has been revised to “More information including model formulas, resultant parameters, and citing references about this model could be found in the section “Materials and methods” of Supplementary Information”, please see Page 8, Lines 154-156.

*5.* *For completeness purposes, please include a comparison of the results reported in this work and others.*

**Response:** Thanks for the comment. A comparison of the results reported in this work and others has been added and listed as follows:

Firstly, the addition of ABC-AQS was found to the favorable for the anaerobic acidogenesis in this study, a comparison of this result and other report has been added, please see Page 11, Lines 215-218;

Secondly, the electron-donating capacity of biochar was increased by 113.5% after the quinone functionalization, this result has been discussed and compared with other work, please see Page 13, Lines 247-251;

Lastly, the methanogenic inhibition by AQS has been focused on and compared to the related works of Zhou et al (2020) and Jiang et al (2022), please see Page 16, Lines 307-312.

*6.* *The authors are advised to briefly address the future perspectives, limitations, and drawbacks of the current study in the conclusion part. It is not necessary to go into great depth when describing the scope of future study; a rough sketch of the areas needing additional research and/or optimization should do.*

**Response:** Thanks for the comment. In this study we hypothesized that the redox activity of biochar could be enhanced by immobilizing quinoid functional groups on its surface, and result showed that the redox activity of biochar was significantly after modification, especially the electron donating capacity of biochar was increased by 113.5% compared to the raw biochar. It was observed that the acidogenesis during anaerobic digestion benefited from the enhanced redox activity of biochar.

However, the methanogenesis of anaerobic digestion was found to be inhibited by the modified biochar. Combining the experimental phenomena in this study and previous works, we believed that the related mechanisms originated from the strong acid treatment stage involved in the biochar modification process. Compared to the improvement of redox activity after biochar modification, the microbial inhibition caused by side reactions during chemical modification may have a stronger impact on anaerobic methanogenesis. The findings of this study give us an important clue that when designing a biochar modification procedure for a given biotechnological process, the possible influences of chemical side reactions during biochar modification process on subsequent microbial metabolism should be emphasized.

Based on the above consideration, the future perspectives and the implications of this work has been added and revised in the section “Results and Discussion” and “Conclusion”, pleased see Page 14, Lines 278-282; Page 16, Lines 320-322; Page 17, Lines 332-334.

*7.* *Please provide a descriptive supporting information section in the manuscript with at least 2-3 sentences.*

**Response:** Thanks for the comment. Done, the description of Supporting Information in the manuscript has been added and revised in the section “Materials and Methods”, please see page 5, lines 88-90; Pages 7, Lines 117-119; Page 8, Lines 154-156.

**Other Revisions:**

In addition to the comments from reviewers, all the other errors we known have been revised throughout the manuscript. To make it clear, the revisions were listed as follows:

| **No.** | **Before revision** | **After revision** | **Page - Line** |
| --- | --- | --- | --- |
| 1 | redox property of biochar was | redox properties of biochar were | Page 2, Line 18 |
| 2 | achieved | reached | Page 2, Line 24 |
| 3 | In the anaerobic digestion test, the acidogenesis rate was improved by 26.3% with the addition of ABC-AQS | The acidogenesis rate was improved by 26.3% with the addition of ABC-AQS during anaerobic digestion test. | Page 2, Line 24-25 |
| 4 | modification process | biochar modification process | Page 2, Line 26 |
| 5 | and | and/or | Page 2, Line 27 |
| 6 | mature organic waste management technology | mature technology for the treatment of organic waste | Page 3, Line 34 |
| 7 | simultaneous organic waste reduction | a combination of waste reduction | Page 3, Line 37 |
| 8 | redox property was | redox properties were | Page 3, Line 42 |
| 9 | greater | higher | Page 3, Line 49 |
| 10 | However, the raw biochar could be modified to the engineered biochar using a variety of physical and chemical techniques, including porosity activation, surface doping, and surface oxidation, to improve the performance of biochar for applications. | However, raw biochar could be modified to improve its performance for applications, using a variety of physical and chemical techniques, including porosity activation, surface doping, and surface oxidation. | Page 3-4, Line 53-55 |
| 11 | were enhanced | were all reported to be enhanced | Page 4, Line 58 |
| 12 | Moreover, | Added | Page 4, Line 59 |
| 13 | and the prepared modified biomass char materials significantly promoted the reduction of organic Congo red removal | then the reduction and the removal of Congo red in the wastewater were significantly promoted by this modified char material. | Page 5, Line 61-62 |
| 14 | served as | was set to | Page 5, Line 93-94 |
| 15 | a | Deleted | Page 7, Line 122 |
| 16 | 37 °C | 37 ± 1 °C | Page 7, Line 124 |
| 17 | experimental reactors | the control and the experimental reactors, respectively. | Page 7-8, Line 137-138 |
| 18 | digestion was repeated | digestion test was operated | Page 8, Line 146-147 |
| 19 | the ZnCl_2_-HCl solution immersion was used as the pre-treatment to enhance the surface immobilization of AQS on ABC | ABC was pretreated by the ZnCl_2_-HCl solution immersion to enhance the surface immobilization of AQS | Page 9, Line 174-175 |
| 20 | on | of | Page 10, Line 179 |
| 21 | rinsed by distilled | rinsed by distilled until pH neutral | Page 10, Line 187 |
| 22 | Added | It has been widely accepted that the anaerobic digestion performance could be improved by the addition of biochar | Page 10-11, Line 196-197 |
| 23 | Added | rapid | Page 11, Line 208 |
| 24 | quinone-imblizalized | quinone functionalized | Page 13, Line 259 |
| 25 | The prolonged strong acid soaking process | The concentrated acid treatment process | Page 15, Line 299 |
| 26 | stage | Deleted | Page 17, Line 327 |
| 27 | has | have | Page 17, Line 328 |
| 28 | production | generation and/or dissolution | Page 17, Line 329 |
